# Notes and Notices

**Published:** 1894-12

**Authors:** 


### NOTES AND NOTICES.

The Metropolitan Post-Graduate School of Medicine.—The demand for Post-Graduate instruction in Homœopathic Materia Medica and Therapeutics has led to the establishment of the Metropolitan Post-Graduate School of Medicine, 245 East Eighty-fourth Street, New York city. It has been found expedient to add to the course in Therapeutics instruction in every branch of Medicine and Surgury by specialists of acknowledged skill and reputation.

The method to be pursued will comprise, first, a thorough examination of the patients by the several specialists in the various departments, who will explain to the class the methods to be followed to make a perfect diagnosis; an explanation of the etiology and pathology of the case, and, when necessary, an illustration of the Hygienic, Dietetic, Mechanical or surgical treatment required.

The specialists in Homœopathic Therapeutics will then select from the other departments such cases as will best illustrate the selection of and results from the exhibition of the homœopathic remedy in all forms of disease.

The Faculty comprises teachers connected with all the Homœopathic Colleges and Hospitals in New York city, and while the various institutions will be drawn upon for clinical material, a nucleus has already been formed by the acquisition of the large general Clinic of the Yorkville Homœopathic Dispensary.

The building is suitably located and well adapted for the purposes of the school.

The system of instruction will be clinical, diseased conditions being demonstrated by the actual examination of the patient, and text-books will not be required.

The fall and winter session will begin on November 15th, and continue until June 1st. The Summer Course will be given during the months of June, July, August, and September. During the Summer Course, the teaching in many of the departments will be chiefly by the instructors, and the fees will be about one-half those of the Winter Session, as will be seen by reference to the fee list.

The Directors wish to emphasize the fact that there will be no private courses, with extra fees attached. No charges will be made for instruction in any branch other than those laid down in the schedule of fees.

The Faculty is very large. No less than sixty-eight professors are on the list. For circulars and special information apply to Charles Deady, M. D., Secretary of the Faculty, 110 West Forty-eighth Street, New York city.

THE LATE DR. LUCIEN B. WELLS left quite a large library of homœopathic books. A list of them has been published in the advertising pages of THE HOMŒOPATHIC PHYSICIAN for the last three months. Physicians and students would do well to secure these books. Address, Mr. E. H. Wells, 31 Summit Place, Utica, N. Y.

"THE HOMŒOPATHIC SOCIETY OF CHICAGO," writes Dr. L. D. Rogers, of *The People's Health Journal*, "is the most flourishing society in Chicago. It represents all the homœopathic schools here. At the October meeting fully one hundred and fifty physicians were present. At the meeting last Wednesday night (November 7th) about seventy-five were present notwithstanding the weather was exceedingly unpleasant."

BILE PIGMENT.—A delicate test for the demonstration of bile pigment in the urine is described in the Toledo *Medical Journal*. After comparing the different tests for the demonstration of bile pigment in the urine, and showing their lack of delicacy in doubtful cases, Dr. Henry Rocin presents a method which has been tried by himself in Prof. Senator's clinic.

He adds ten parts of the official tincture of iodine to ninety parts of alcohol, which mixture is kept ready for use. A sample of urine to be examined is poured into a test-tube, which, being held inclined, has from 2-3 ccm. (30–45 min.) of the above dilute tincture poured upon it with great care, so that the same rests upon the urine without mingling with it. Almost instantly, at the plane of contact of the two fluids, a grass-green ring is developed, which oftentimes persists for hours. If there is no bile pigment present, the yellow urine has either only a light yellow or colorless ring formed at the meeting of the two solutions.

This test has been used for three-fourths of a year at Prof. Senator's clinic, and, after comparing it with a large variety of tests, it has been demonstrated as the most delicate, most reliable, and simplest test for the detection of bile pigment.—*Medical Examiner*.

MORBID CONDITIONS OF THE HEART.—Dr. W. C. Cahall mentions the morbid conditions of the heart and the evils to be feared in a recent number of the *American Medico-Surgical Journal*.

| DISEASE | EVILS TO BE FEARED |
| --- | --- |
| Simple Dilatation, | Muscle weak and yielding from increased intra-ventricular pressure during diastole. |

| | |
|---|---|
| Simple Hypertrophy, | Over-developed and over-acting muscle. Increased vigor and frequency of contraction, and increased arterial tension. |
| Aortic Stenosis, | Increased intra-ventricular pressure from excess of blood retained and inflowing from auricle during diastole, venous engorgement and lessened arterial tension. |
| Aortic Regurgitation, | Passive dilatation of left ventricle, the result of regurgitating blood during diastole. Lowered arterial tension resulting in ill-nourished heart muscles. |
| Mitral Stenosis, | Distention and dilatation of left auricle from inability to empty itself during diastole. Venous engorgement and lowered arterial tension. |
| Mitral Regurgitation, | do. do. do. together with a dilated hypertrophy of the left ventricle. |
| Tricuspid Regurgitation, | Distention and dilatation of right auricle from regurgitation of blood. Venous stasis. |

—*Medical Examiner.*

---

### FUN FOR DOCTORS.

### CURE FOR CORNS.

Riley's charm for the cure of corns is a receipt well worth knowing, and it is perhaps interesting, too, as a bit of Hoosier folklore:

Prune your corn in the gray of the morn
With a blade that's shaved the dead,
And barefoot go, and hide it so
The rain will rust it red;
Dip your foot in the dew, and put
A print of it on the floor,
And stew the fat of a brindle cat,
And say this o'er and o'er:
Corny! morny! bady! dead!
Gorey! sorey! rusty! red!
Footsy! putsy! floory! stew!
Fatsy! catsy!
Mew!
Mew!
Come, grease my corn
In the gray of the morn!
Mew! mew! mew!

—*Philadelphia Times.*

